# Radiofrequency Transoral Microsurgical Procedures in Benign and Malignant Laryngeal and Hypopharyngeal Lesions (Institutional Experiences)

**DOI:** 10.1155/2015/926319

**Published:** 2015-02-18

**Authors:** Krisztina Somogyvári, Imre Gerlinger, László Lujber, András Burián, Péter Móricz

**Affiliations:** ^1^Department of Otorhinolaryngology, Head and Neck Surgery, Medical School, University of Pécs, Munkácsy M. Street 2, Pécs 7621, Hungary; ^2^Department of Otorhinolaryngology, János Balassa Hospital, Béri B. Á. Street 5-7, Szekszárd 7100, Hungary

## Abstract

Besides cold-steel and laser instruments, the use of radiofrequency (RF) devices in transoral microsurgery is getting increasing popularity mainly due to its minimal thermal effect on the collateral soft tissue. Authors summarize their surgical technique, results, and experience gained with RF applied during laryngeal interventions at the Department of Otorhinolaryngology, Head and Neck Surgery at Medical School, University of Pécs. Transoral microsurgery using radiofrequency was carried out in 23 cases in total between 1 January 2011 and 1 March 2013. Fourteen histopathologically different benign lesions and 9 malignant planocellular carcinomas of the larynx were removed using different Micro-Larynx RF Probes powered by Surgitron Dual 4.0 MHz Frequency RF (Ellman International, Oceanside, NY, USA) device. No major bleeding event occurred during or after the procedures and neither laryngeal oedema nor significant postoperative pain was recorded. Authors also reviewed the international literature in this topic while detailing some of their most interesting cases.

## 1. Introduction

Besides traditional cold-steel and CO_2_ laser instruments, interventions using radiofrequency (RF) devices are becoming more popular in transoral microsurgery [1]. There are several reasons in the background of their becoming more and more widespread. On the one hand, radiofrequency devices produce less collateral heat, thus less adjacent tissue damage and scarring. On the other hand, this method also provides the benefits of the effects of laser in bleeding control [2].

In the present paper authors provide details and analyse experiences gained with radiofrequency transoral microsurgical procedures performed at the Department of Otorhinolaryngology, Head and Neck Surgery at the Medical School, University of Pécs.

## 2. Patients and Methods

Transoral microsurgeries using radiofrequency were carried out at our department in 23 cases between 1 January 2011 and 1 March 2013, in 14 cases due to benign and in 9 cases due to malignant histopathological lesions. In the case of two of our patients who had previously undergone partial larynx resection and radiation therapy, the method was applied to facilitate scar formation of the extremely oedemic mucosa covering the arytenoid region.

All surgeries were performed under intratracheal narcosis. The thinnest possible tube was used for intubation; in one case JET ventilation was applied (TwinStream, Carl Reiner GmbH, Vienna, Austria).

Micro-Larynx RF Probes powered by Surgitron Dual 4.0 MHz Frequency RF (Ellman International, Oceanside, NY, USA) were used with needle-tip and ball-tip electrodes.

Needle-tip electrodes enable precise surgical incision making, while ball-tip electrodes ensure focused electrocoagulation.

## 3. Results

Table 1 summarises our experiences and findings regarding histologically benign lesions.

RF ablation carried out on the arytenoid mucosa made swallowing easier in both patients. A rare disorder was seen in a 57-year-old male patient, a lipoma causing the prominent vestibular fold on the right side and thereby a constant feeling of a lump in the throat and difficulty swallowing. Two other interesting and rare cases were those of two patients with isolated laryngeal amyloidosis. Systemic amyloidosis was excluded in both cases with clinical examinations. The 51-year-old woman and the 32-year-old man were both complaining of temporary hoarseness and the feeling of a lump in the throat caused by the protruding extra tissue on the vestibular fold which proved to be amyloidosis and which was consequently removed.

In the case of a 68-year-old formerly laryngectomised patient, the 1 cm diameter, sessile mucosal protrusion at the oesophageal entrance preventing the retrograde retraction of the voice prosthesis was found during oesophagoscopy performed as the initial step of the first voice prosthesis implantation.

Six weeks after the removal of the mass histologically diagnosed as a polyp, the voice prosthesis could be implanted without difficulty.

In one case, in consequence of permanent intubation, tracheal stricture occurred anteriorly at the level of the first tracheal cartilage. Beside JET ventilation the web narrowing the lumen was resected with the needle-tip electrode. Neither dilatation nor mitomycin was applied, and any stenosis appeared postoperatively.

All lesions characterised by malignant histological changes proved to be planocellular carcinomas by histological examination. Tumours of the vocal cord and T1 tumours of the hypopharynx were removed with R0 resection.  Table 2 summarises our findings.

In the case of vocal cord tumours, the tumours could be excised fast and with adequately delicate movements in 2 patients as primary therapy and in another 2 patients following radiation therapy. It is of note that in the case of a 92-year-old postradiotherapy patient with the help of the newly acquired JET ventilation device it was possible to completely remove the recidivation in the posterior third of the vocal cord by eliminating the space reduction of the intubation tube.

In the case of two of our patients with T1 tumours of the hypopharynx, excisions were easier due to the pedunculated nature of the tumours. Following radiotherapy, the approximately 0.5 cm diameter exulcerated tumour residuum was located in between the anterior and medial walls of the recessus piriformis, and by a resection with a free margin the patient did not require laryngectomy.

In those cases where debulking of the T3, supraglottic-origin tumours was carried out, debulking was always effective and tracheotomy was not required prior to later laryngectomies in any of the more advanced cases.

The insignificant amount of bleeding during the surgeries did not interfere with precise excising. Practically, irradiated, scarred areas could be excised without bleeding. The postoperative period was without the development of laryngeal oedema or significant pain in all patients.

At the first postoperative examination reepithelisation was found to be in a more advanced stage than it would be with the use of cold-steel or laser devices. Although this is merely a subjective statement, at the second postoperative examination reepithelisation—except for the tumour debulking cases—was complete.

## 4. Discussion

Two important requirements should be fulfilled for surgeons during transoral microsurgical interventions:

- a precise surgical incision with satisfying special controllability,
- effective bleeding-control with the least possible tissue damage.

The traditional cold-steel technique meets the first criterion; however, our experiences showed that bleeding during excisions disturbs evaluation of margins; moreover, heat from later electrocoagulation can be quite significant. Ragab et al. used RF and cold-steel methods randomly in 50 cases of benign vocal cord lesions [1]. As a conclusion, there was no difference between the two groups in duration of surgery, postoperative events, and pain ratings. Restivo et al. carried out RF excisions of 18 glottic tumours via cordectomy, bilateral cordectomy, or bilateral extended cordectomy procedures [2]. In our study according to the histopathological findings the resection margins were clear in all cases except for tumour debulking. Preparations could be carried out practically without any bleeding. Postoperative oedema developed in only an insignificant number of cases, pain or dysphagia did not occur, and hospitalisation time decreased significantly.

In the case of CO_2_ laser surgeries good results can be expected both in terms of precise incision making and effective bleeding control. One disadvantage can be due to the spreading features of the CO_2_ laser beam; namely, the possibility to curve an incision spatially is limited. Another disadvantage is that devices are more costly. Special devices and much more expensive laser intubation tubes are required to prevent accidents caused by the laser beam. Whereas the former is a one-time investment, the latter increases the costs of every intervention using laser. The use of JET ventilation allows better access to the surgical area and the use of the laser tube can also be spared. Based on experience gained with RF removal of 44 glottic and supraglottic tumours Alberdi et al. state that there is no difference between CO_2_ laser excision and radiosurgery concerning local tumour control, complications, and morbidity but claim that the RF method is more cost-effective [3].

**Figure 1:**
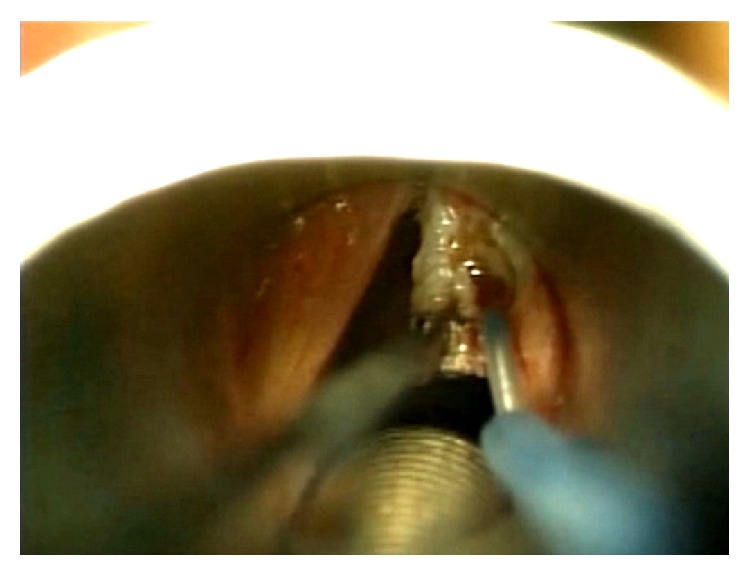
Excision of a right vocal cord pachydermia with a needle-tip RF electrode.

Besides less collateral heat, RF excision provides all advantages of CO_2_ laser surgery. It is also easier to manipulate the excision line in space as a result of the bayonet-shaped tip of the device which makes the tip visible throughout the surgical procedure (Figure 1).

Our experiences showed that this “shoulder” of the device may get stuck in some marginal positions and thereby may make excision and surgical manipulation more difficult. This method does not require any special complementary devices. Through research using animal models, Divi et al. found that the vocal cord of a dog completely reepithelised by day 7, the muscles of the cord were not damaged, and as compared to the CO_2_ laser procedure studied earlier the extent of inflammatory response was also reduced [4]. Our experiences are in agreement with the above findings. Due to less collateral heat and therefore better margin control, radiofrequency provides more precise incision line in comparison to CO_2_ laser excision.

In the present study, similar to international experience and findings, precise surgical excisions and precise bleeding control could be achieved with minimal bleeding in the majority of cases. In the postoperative period neither laryngeal oedema nor intense pain occurred. Reepithelisation was faster than it usually is in the case of cold-steel or CO_2_ laser procedures.

Radiofrequency is becoming more and more widespread in transoral microsurgical procedures. Katona et al. published his findings with using a RF loop electrode in aryepiglottoplasty of two children with stridor whose previous polysomnographies proved deep nocturnal apnoe and episodes of hypoxia. Further indications were the children getting tired during feeding and slow weight gain [5]. In a later publication, Katona provides a review of the use of RF in otorhinolaryngology [6].

In international literature, in paediatric surgery Srivastava used the method successfully in the treatment of laryngomalacia [7], Gonik and Smith in the treatment of a vallecular cyst [8], and Kumar et al. in the removal of a laryngeal cyst [9]. In adductor spasmodic dysphonic microlaryngeal RF ablation was carried out by Remacle et al. in unilateral recurrent nerve [10] and by Kim et al. on bilateral thyroarytenoid muscles [11]. Timms et al. performed it in laryngeal [12] while Carney et al. in tracheolaryngeal papillomatosis; the latter authors found it as a direct alternative to CO_2_ laser [13].

## 5. Conclusion

Radiofrequency incorporates the advantages of both CO_2_ laser and cold-steel techniques; it provides precise incision line, good haemostasis, and fast reepithelisation and is a cost-effective transoral microsurgical procedure.

## Figures and Tables

**Table 1:** Histologically benign lesions and our findings.

Histologically benign lesions	Number of cases	Notes
Unilateral vocal cord pachydermia	4	Following irradiation, 1 hypopharynx, 1 vocal cord tumour

Unilateral vocal cord polyp	2	1 sessile, 1 pedunculated

Laryngeal papilloma	1	Monolateral vocal cord and anterior commissure localisation, complete removal

Laryngeal amyloidosis	2	Isolated amyloidosis on the vestibular fold

Laryngeal lipoma	1	On the right vestibular fold

Polyp at the oesophageal entrance	1	Following laryngectomy it disturbed implantation of the voice prosthesis

Tracheal stricture	1	At the level of the first tracheal cartilage

Postirradiation oedema of the laryngeal entrance	2	Focused treatment of the arytenoid area to improve swallowing

**Table 2:** Histologically malignant lesions and our findings.

Histologically malignant cases	Number of cases	Notes
Hypopharyngeal tumour (T1)	3	2 primary surgeries, 1 surgery following irradiation

Vocal cord tumour (T1)	4	2 primary surgeries, 2 surgeries following irradiation (1 located at the posterior third of the vocal cord, JET ventilation)

Supraglottic tumour (T3)	2	Tumour debulking to avoid tracheotomy
